# Development and validation of the Amyloidosis Area and Severity Index for primary cutaneous amyloidosis

**DOI:** 10.1016/j.jdin.2026.09.009

**Published:** 2026-10-01

**Authors:** Ziqi Jiang, Siru Wang, Jianxia Xiong, Shilin Chen, Chengzhi Li, Zelong Li, Yun Pan, Chuan Liu, Rentao Yu, Qian Liu, Yujing Huang, Songyao Deng, Fengzeng Li, Aijun Chen, Wenyan He

**Affiliations:** Department of Dermatology, The First Affiliated Hospital of Chongqing Medical University, Chongqing, China

**Keywords:** AASI, Delphi consensus, primary cutaneous amyloidosis, reliability, severity instrument, validity

## Abstract

**Background:**

Primary cutaneous amyloidosis (PCA) is a chronic, pruritic skin disorder lacking a standardized severity instrument, hindering clinical assessment and research.

**Objectives:**

To develop and validate a novel clinician-reported outcome measure, the Amyloidosis Area and Severity Index (AASI), for PCA.

**Methods:**

The AASI was developed via a modified Delphi consensus process informed by surveys of 340 patients and 33 dermatologists. In a prospective validation study involving 50 patients, 10 raters independently assessed PCA patients twice using the AASI. Interrater and intrarater reliability were determined using intraclass correlation coefficients, and construct validity by correlating AASI scores with established outcome measures.

**Results:**

The Delphi process prioritized 4 core domains: papule/macule, dryness/scaling, excoriation, and lichenification. The AASI demonstrated moderate interrater (intraclass correlation coefficient = 0.701) and good-to-excellent intrarater reliability (intraclass correlation coefficient range: 0.762-0.942). Construct validity was supported by strong correlations with Investigator's Global Assessment (ρ = 0.84) and Xerosis Score (ρ = 0.75) (all *P* < .001).

**Limitations:**

Delphi consensus restricted to Chinese clinicians and patients; single-center validation; treatment responsiveness being evaluated in an ongoing trial.

**Conclusions:**

The AASI is a new, consensus-derived severity instrument for PCA with moderate-to-good reliability and validity, providing a foundation for future standardized assessment for clinical and trial use.

**Capsule Summary**

• This study establishes a novel disease-specific severity instrument for primary cutaneous amyloidosis, developed via a modified Delphi consensus and validated for favorable measurement properties.
• Further validation could support adoption of this tool to facilitate objective monitoring and high-quality trials, addressing a barrier to advancing care for this disease.

## Introduction

Primary cutaneous amyloidosis (PCA) is a chronic pruritic dermatosis characterized by localized amyloid deposition in the skin without systemic involvement.^1^ The 2 major subtypes, lichen amyloidosis (LA) and macular amyloidosis (MA), differ in predominant lesion morphology but share common clinical features, including pruritus, scaling, excoriation, and lichenification resulting from chronic scratching.^2^, ^3^, ^4^ Persistent symptoms and visible skin lesions substantially impair patients' quality of life.^5^

Despite increasing research interest, progress in clinical research has been hindered by the absence of a standardized severity instrument. Current evaluation relies on generic measures, including the Investigator's Global Assessment (IGA), body surface area (BSA), Peak Pruritus Numerical Rating Scale (PP-NRS), and Dermatology Life Quality Index (DLQI),^1^, ^6^ which evaluate individual aspects but none are specific to PCA morphologic manifestations. Consequently, disease severity is assessed inconsistently across clinical practice and research, limiting study comparisons and therapeutic evaluation.

Disease-specific clinician-reported outcome measures (ClinROMs) have transformed outcome assessment in inflammatory skin diseases by providing standardized, reproducible, and clinically meaningful measures of disease severity. Instruments such as the Eczema Area and Severity Index (EASI)^7^ and Psoriasis Area and Severity Index^8^ have become essential endpoints in practice and trials. In contrast, no validated ClinROM currently exists for PCA. Guided by the COnsensus-based Standards for the selection of health Measurement INstruments (COSMIN) framework, developing a robust ClinROM requires systematic evaluation of content validity, reliability, and construct validity.^9^, ^10^ Such an instrument is essential to standardize assessment, facilitate therapeutic evaluation, and improve comparability across future studies.

Accordingly, this study aimed to develop and initially validate the Amyloidosis Area and Severity Index (AASI), the first disease-specific ClinROM for PCA. The AASI was developed through a modified Delphi consensus process incorporating both patient and dermatologist perspectives and subsequently validated by evaluating its interrater and intrarater reliability and construct validity.

## Materials and methods

### Study design

This study was conducted at the Department of Dermatology, the First Affiliated Hospital of Chongqing Medical University, and consisted of 2 sequential phases: (1) instrument development phase: modified Delphi consensus process informed by an initial patient survey and 2 rounds of physician surveys with an interim consensus discussion to develop the AASI; (2) instrument validation phase: a prospective reliability and validity study of the AASI (Fig 1).

**Fig 1:**
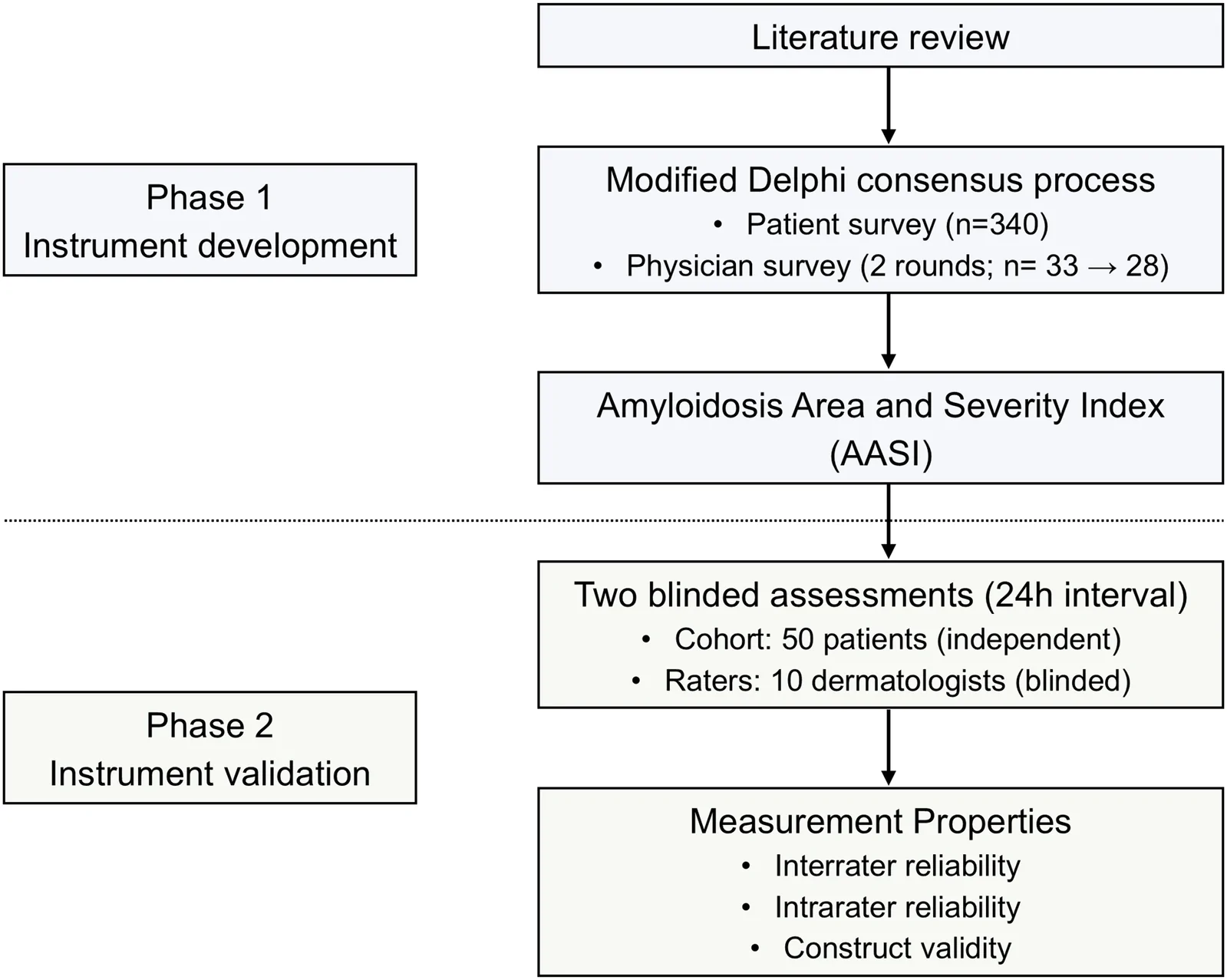
Development and validation workflow of the AASI for primary cutaneous amyloidosis. Validation cohort and raters were independent of instrument development. *AASI*, Amyloidosis Area and Severity Index.

### Phase 1: Instrument development

#### Preliminary patient survey

Initial AASI candidate items were derived from a targeted review of PCA phenotypic literature, then embedded in uniform clinician/patient questionnaires (differing only in demographic modules). A total of 340 patients clinically and/or histologically diagnosed with PCA were enrolled. To identify the most representative manifestations, all participants completed structured questionnaires on common PCA manifestations, with multiple selections permitted (Supplementary Table I, available via Mendeley at https://data.mendeley.com/datasets/4mr99jp5r4/1; definitions therein).

#### Modified Delphi consensus process

The expert panel comprised 33 dermatologists with recognized clinical/academic expertise in PCA, purposively recruited from tertiary medical centers across 5 Chinese provinces/municipalities (Supplementary Methods, available via Mendeley at https://data.mendeley.com/datasets/4mr99jp5r4/1). Eligibility was confirmed through self-reported clinical and academic experience collected in the baseline survey. Following the initial physician survey (Supplementary Table II, available via Mendeley at https://data.mendeley.com/datasets/4mr99jp5r4/1), aggregated findings from both surveys were presented at a structured virtual consensus meeting (Supplementary Methods, available via Mendeley at https://data.mendeley.com/datasets/4mr99jp5r4/1). A second-round survey was subsequently completed by 28 experts (84.8% retention). Consensus was predefined as ≥70% agreement (agree or strongly agree). Open-ended items invited additional manifestations in both the patient and physician questionnaires. The AASI was developed based on the consensus-selected domains to quantify PCA severity by integrating affected BSA and lesion severity.

### Phase 2: Instrument validation

#### Patients

Fifty PCA patients were prospectively recruited at our dermatology outpatient clinic from March to August 2025. Sample size aligns with COSMIN reliability study criteria.^11^ Diagnosis was confirmed via clinical evaluation, dermoscopy, and/or skin biopsy. Exclusion criteria were: (1) PCA-directed treatment between assessment day 1/2, preserving scoring consistency; (2) active comorbid skin diseases interfering with scoring; (3) other condition precluding participation. Written informed consent was obtained from all participants.

#### Raters

Ten dermatologists from our department (residents to chief physicians) served as independent raters, receiving the protocol and assessment forms 1 week before validation, followed by standardized 30-minute training and 30-minute discussion. The raters then independently evaluated 4 representative PCA cases in a pilot exercise to familiarize themselves with the procedure and provide structured feedback on relevance, comprehensiveness, and comprehensibility of the AASI. No modifications to the instrument were required based on this feedback. None had participated in the expert panel that developed the AASI, minimizing potential assessment bias.

### Reference assessment tools

To evaluate construct validity, AASI scores were correlated with several established clinical measures:

• IGA: Rater-assigned 5-point scale (0 = clear to 4 = severe) capturing PCA severity based on key morphological features adapted from validated IGA for atopic dermatitis^12^; full criteria in Supplementary Methods, available via Mendeley at https://data.mendeley.com/datasets/4mr99jp5r4/1.
• BSA: Rater-assigned involved BSA estimated using the handprint method (palmar surface ≈ 1% of total BSA).
• Xerosis Score: Rater-assigned 4-point scale (0 = absent to 3 = severe) quantifying the severity of dryness.
• PP-NRS: Mean of daily peak pruritus scores (0-10 scale), each representing worst itch intensity in the past 24 hours, calculated from at least 4 days within the past week.^13^
• DLQI: Patient-reported 10-item questionnaire measuring quality-of-life impact over the previous week (range 0-30).^14^

### Clinical evaluation procedure

All 50 patients, enrolled in 2 batches, underwent assessments on 2 occasions 24 hours apart. In each session, patients were assigned randomized sequences and evaluated independently by all 10 raters. Raters stayed in designated rooms while patients rotated. A trained assistant recorded scores. Session 2 used a new sequence with raters blinded to prior scores. Reference measures were assessed in session 1; AASI was assessed in both sessions. Standardized photographs were obtained before the first assessment.

### Statistical analysis

Reliability was assessed using intraclass correlation coefficient (ICC) (A,1) (2-way mixed-effects model for absolute agreement).^15^ Inter-rater reliability used ICC for both sessions, with session 1 as primary. ICCs were interpreted as: <0.50 poor; 0.50-0.75 moderate; 0.75-0.90 good; >0.90 excellent.^16^ Construct validity was evaluated at the patient level via Pearson (continuous) or Spearman (ordinal) correlations with IGA, BSA, Xerosis Score, PP-NRS, and DLQI, using first-session scores, with clinician-assessed measures averaged across 10 raters and patient reported outcomes, yielding 1 observation per patient. 95% confidence intervals were estimated using bias-corrected accelerated bootstrap (5000 resamples, seed 2026). Correlation strength was interpreted as: 0.00-0.10 negligible, 0.10-0.39 weak, 0.40-0.69 moderate, 0.70-0.89 strong, 0.90-1.00 very strong.^17^ COSMIN-guided prespecified hypotheses are in Supplementary Table VII, available via Mendeley at https://data.mendeley.com/datasets/4mr99jp5r4/1. A *P* value <.05 was significant. Analyses were performed using SPSS 21.0 (IBM Corp.).

## Results

### Phase 1: Instrument development results

#### Patient survey results

Participant characteristics are summarized in Supplementary Table III, available via Mendeley at https://data.mendeley.com/datasets/4mr99jp5r4/1. Among 340 PCA patients, pigment abnormality was most frequent (92.6%, 315/340), followed by lichenification (79.7%, 271/340), macule (78.2%, 266/340), papule (77.6%, 264/340), excoriation (67.6%, 230/340), and dryness/scaling (65.6%, 223/340). Nodule, erythema, and other manifestations were less commonly reported (Supplementary Table IV, available via Mendeley at https://data.mendeley.com/datasets/4mr99jp5r4/1). Free-text responses from both patients and physicians yielded no novel, clinically distinct skin manifestations beyond the predefined item pool, which therefore remained unchanged.

#### Expert consensus on core clinical features

In the initial survey, 33 dermatologists independently rated clinical relevance. The most selected features were lichenification and papule (75.8%, 25/33), followed by macule (66.7%, 22/33), pigment abnormality (39.4%, 13/33), nodule (39.4%, 13/33), dryness/scaling (36.4%, 12/33), and excoriation (27.3%, 9/33) (Table I). Following group discussion, 28 of the original 33 dermatologists completed round-2 voting, with increased consensus for dryness/scaling (75.0%, 21/28) and excoriation (71.4%, 20/28), and sustained high agreement for macule (100.0%, 28/28; consensus-endorsed MA hallmark), papule (96.4%, 27/28; consensus-endorsed LA hallmark), and lichenification (92.9%, 26/28) (Table I). Pigment abnormality fell to 17.9% (5/28) and was ultimately excluded as consensus held it persists post-improvement, poorly reflecting inflammatory activity. Nodule and erythema were also excluded due to low frequency and limited applicability across PCA subtypes.

**Table I:** Expert consensus on core clinical features of primary cutaneous amyloidosis via modified Delphi process

Manifestation	Count (%)	
	Delphi round 1 (*n* = 33)	Delphi round 2 (*n* = 28)
Papule	25 (75.8)	27 (96.4)
Macule	22 (66.7)	28 (100.0)
Lichenification	25 (75.8)	26 (92.9)
Excoriation	9 (27.3)	20 (71.4)
Dryness/scaling	12 (36.4)	21 (75.0)
Pigment abnormality	13 (39.4)	5 (17.9)
Nodule	13 (39.4)	7 (25.0)
Erythema	4 (12.1)	1 (3.6)

Data are presented as *n* (%).

#### Final AASI instrument

Based on consensus results, 4 core domains were selected: papule/macule, dryness/scaling, excoriation, and lichenification. Papule and macule were combined into 1 domain because they correspond to the 2 major PCA subtypes (LA and MA), whereas the other 3 domains are shared across both subtypes. The AASI quantifies PCA severity via affected BSA and 4 core clinical signs above. BSA involvement across 4 anatomical regions (reflecting standard BSA proportions per EASI/Psoriasis Area and Severity Index and ratified by Delphi consensus, head and neck: 10% weight; upper extremities: 20%; trunk: 30%; lower extremities: 40%) is scored on a 7-point ordinal scale (0 = 0%; 1 = <10%; 2 = 10% to 29%; 3 = 30% to 49%; 4 = 50% to 69%; 5 = 70% to 89%; 6 ≥ 90%). Each sign is graded per region on a 0-3 scale (0 = none, 1 = mild, 2 = moderate, 3 = severe; definitions in Supplementary Table V, available via Mendeley at https://data.mendeley.com/datasets/4mr99jp5r4/1). Total AASI (range 0-72) sums regional scores: (sum of sign scores) × BSA score × regional weight (Table II).

**Table II:** Final AASI scoring sheet

Item	Head and neck	Upper limbs	Trunk	Lower limbs
Papule/macule (0-3)				
Dryness/Scaling (0-3)				
Excoriation (0-3)				
Lichenification (0-3)				
Regional sign subtotal (0-12)				
BSA involvement score (0-6)				
Regional weight	×0.1	×0.2	×0.3	×0.4
Regional score				
Total AASI (0-72)				

Sign scores: 0 = none, 1 = mild, 2 = moderate, 3 = severe (detailed definitions in Supplementary Table V, available via Mendeley at https://data.mendeley.com/datasets/4mr99jp5r4/1).

BSA involvement score: 0 = 0%, 1 = <10%, 2 = 10% to 29%, 3 = 30% to 49%, 4 = 50% to 69%, 5 = 70% to 89%, 6 = ≥90%.

Calculation: Regional score = (sum of 4 sign scores, 0-12) × regional BSA score (0-6) × regional weight. Total AASI = sum of the 4 regional scores (range 0-72).

Anatomical definitions: Buttocks and feet were rated as part of the lower extremities; internal axillae and groin as part of the trunk; external axillae and hands as part of the upper extremities. Half-points are permitted (1.5 and 2.5); 0.5 is not allowed.

*AASI*, Amyloidosis Area and Severity Index; *BSA*, body surface area.

### Phase 2: Instrument validation results

#### Patient characteristics

Fifty PCA patients distinct from the 340 developmental-survey participants were enrolled, comprising 32/50 males (64.0%) and 18/50 females (36.0%), aged 20-77 years. Disease subtypes included LA (14/50, 28.0%), MA (30/50, 60.0%), and mixed-type (LA + MA; 6/50, 12.0%), differing from the 45.9% mixed type in the preliminary survey due to self-reporting versus physician-confirmed subtyping. Disease duration ranged from 0.3 to 35 years. 31 (62.0%) of them were skin biopsy-confirmed, and 19 (38.0%) via dermoscopy plus clinical evaluation (Table III).

**Table III:** Demographic and clinical characteristics of the validation cohort (*n* = 50)

Characteristic	Values
Age (y)	44.5 (20-77)
Sex (male/female)	32 (64.0%)/18 (36.0%)
Disease subtype (LA/MA/mixed [LA + MA])	14 (28.0%)/30 (60.0%)/6 (12.0%)
Disease Duration (y)	8.0 (0.3-35.0)
Diagnostic confirmation (skin biopsy/dermoscopy)	31 (62.0%)/19 (38.0%)

Data are presented as median (range) or *n* (%).

*LA*, Lichen amyloidosis; *MA*, macular amyloidosis.

#### Interrater reliability of AASI

The AASI demonstrated moderate overall interrater reliability in the first session (ICC = 0.701; 95% confidence interval, 0.564-0.807), improved upon re-assessment 1 day later (ICC = 0.744; 95% confidence interval, 0.639-0.831). Stratified analyses revealed good interrater reliability in LA (ICC = 0.760) and mixed (ICC = 0.750), and moderate in MA (ICC = 0.659). By body region, interrater reliability was moderate for head and neck, upper extremities, and trunk, and good for lower extremities (Table IV). For clinical signs, ICCs ranked papule/macule, lichenification, then dryness/scaling and excoriation (Supplementary Table VI, available via Mendeley at https://data.mendeley.com/datasets/4mr99jp5r4/1). Second-session ICCs are provided in Supplementary Table VI, available via Mendeley at https://data.mendeley.com/datasets/4mr99jp5r4/1, with most values slightly higher than the first session.

**Table IV:** Interrater and Intrarater Reliability of the AASI

Assessment	Interrater ICC	Interrater reliability∗	Intrarater ICC	Intrarater reliability∗
Overall	0.701 (0.564-0.807)	Moderate	0.872 (0.762-0.942)	Good
By subtype				
LA	0.760 (0.577-0.899)	Good	0.900 (0.782-0.985)	Good
MA	0.659 (0.487-0.800)	Moderate	0.873 (0.618-0.932)	Good
Mixed (LA + MA)	0.750 (0.496-0.950)	Good	0.926 (0.485-0.984)	Excellent
By body region				
Head and neck	0.510 (0.402-0.631)	Moderate	0.861 (0.620-0.963)	Good
Trunk	0.732 (0.626-0.821)	Moderate	0.880 (0.721-0.953)	Good
Upper extremities	0.710 (0.607-0.802)	Moderate	0.868 (0.681-0.909)	Good
Lower extremities	0.813 (0.736-0.877)	Good	0.934 (0.872-0.961)	Excellent

*AASI*, Amyloidosis Area and Severity Index; *ICC*, intraclass correlation coefficient; *LA*, lichen amyloidosis; *MA*, macular amyloidosis.

∗ All interrater ICCs represent the first assessment session and are presented as ICC (95% confidence interval) (see Supplementary Table VI, available via Mendeley at https://data.mendeley.com/datasets/4mr99jp5r4/1 for Session 2 results); intrarater ICCs are reported as median (range) across 10 raters. ICC values are interpreted as follows: <0.50 poor; 0.50-0.75 moderate; 0.75-0.90 good; >0.90 excellent.

#### Intrarater reliability of AASI

Overall intrarater reliability ranged from good to excellent, with ICC values between 0.762 and 0.942 across all raters. Eight of 10 raters exhibited ICCs above 0.85. By subtype, intrarater reliability was consistently high across all categories, with median ICC of 0.900 for LA, 0.873 for MA, and 0.926 for mixed. By body region, lower extremities showed the highest intrarater reliability (median ICC = 0.934), while head and neck demonstrated the lowest (median ICC = 0.861) (Table IV).

#### Construct validity of AASI

AASI scores showed an expected strong correlation with BSA (Pearson's r = 0.86, *P* < .001). Strong positive correlations were observed with IGA (Spearman's ρ = 0.84, *P* < .001) and Xerosis Score (Spearman's ρ = 0.75, *P* < .001). In contrast, PP-NRS showed a weak positive correlation (Spearman's ρ = 0.34, *P* < .05), while DLQI showed no significant correlation (Spearman's ρ = 0.00, *P* = .99) (Supplementary Table VII, available via Mendeley at https://data.mendeley.com/datasets/4mr99jp5r4/1). Stratified analysis by PCA subtype is presented in Supplementary Table VII, available via Mendeley at https://data.mendeley.com/datasets/4mr99jp5r4/1.

## Discussion

This study developed and initially validated the AASI, the first disease-specific ClinROM for PCA. Developed per COSMIN framework, the AASI demonstrated moderate-to-good reliability and construct validity, offering an objective severity assessment tool. As an initial validation study, these findings support its further evaluation in larger cohorts and establish a foundation for standardized assessment in clinical practice and future trials.

A major strength of the AASI is its rigorous, disease-specific development via a modified Delphi process incorporating both clinician and patient perspectives, ensuring content validity.^18^ Although pigmentary change was highly prevalent and prioritized by patients for cosmetic reasons, clinicians regarded it as a relatively stable sequela rather than an indicator of current disease activity. Its inclusion would therefore impair responsiveness, as pigmentary abnormalities frequently persist despite clinical improvement. Accordingly, the AASI was designed to capture reversible, treatment-responsive disease activity rather than cumulative damage, with cosmetic and quality-of-life impacts better assessed by complementary PROs. Furthermore, broad consensus that LA and MA share core morphologic domains supported the development of a single instrument applicable across the PCA spectrum, facilitating standardized outcome assessment in both routine practice and future clinical trials.^19^

Unlike generic measures (eg, IGA and BSA), the AASI integrates disease-specific domains with BSA, paralleling EASI/Psoriasis Area and Severity Index but tailored to PCA.^8^, ^20^ Domains were equally weighted to optimize simplicity, usability, and reproducibility. Construct validity was supported by strong IGA and xerosis correlations. The strong BSA correlation was expected, as BSA is an AASI component. Subgroup analysis showed consistent construct validity across PCA subtypes. The weak correlation with PP-NRS and negligible correlation with DLQI suggest that morphological severity, symptom burden, and quality of life reflect different dimensions of disease burden and may not vary in parallel, particularly in the context of this chronic disease. Given the partial overlap in content among measures and use of the same rater for certain assessments, these correlations should be interpreted as supportive rather than definitive evidence of convergent validity. Overall, these findings support the AASI as a standardized, disease-specific measure of morphologic severity that complements patient-reported assessments of symptoms and quality of life.

The AASI also demonstrated favorable measurement properties, with moderate-to-good interrater and intrarater reliability across clinicians following standardized training, supporting its suitability for multicenter and longitudinal studies. Stratified analyses revealed lower reliability for head/neck, likely reflecting anatomical complexity, hair obstruction, and greater variability in scoring dryness/scaling and excoriation than papule/macule and lichenification. These findings probably reflect greater subjectivity in evaluating dynamic clinical features with xerosis and scratching.^7^ Similar regional or feature-specific variability has been reported for other instruments, including EASI and the Vitiligo Area and Severity Index.^21^ Importantly, overall reliability remained stable across subtypes and presentations, and improved slightly in the second session, indicating these variabilities did not materially compromise instrument performance and brief familiarization enhanced scoring consistency. These findings highlight areas where focused training may improve consistency, particularly in multicenter trials requiring measurement precision.

Several limitations should be acknowledged. First, the Delphi panel lacked international participation despite including multi-regional Chinese experts, warranting cross-cultural validation. Second, single-center recruitment and modest sample size of the validation cohort may limit generalizability, though meeting reliability recommendations. Third, responsiveness, measurement error, minimal clinically important differences, and structural validity were not evaluated; however, an ongoing trial (NCT07143864) is assessing AASI responsiveness. Fourth, moderate absolute agreement between raters indicates some variability in scoring, suggesting that further rater standardization and training may improve scoring consistency. Finally, future studies using independent raters may provide further support for construct validity. Future multicenter studies should address these gaps by performing factor analysis, evaluating responsiveness, establishing clinically meaningful severity categories and responder definitions, and facilitating its application as a standardized trial outcome.

## Conclusions

In conclusion, the AASI is a novel, consensus-derived, disease-specific ClinROM that addresses the unmet need for standardized assessment in PCA. Developed and validated per COSMIN guidelines, it is feasible, requires only minimal training, and demonstrates moderate-to-good reliability and validity. This initial validation study presents the AASI as a promising new instrument, with ongoing research validating its responsiveness to treatment to facilitate its adoption as a trial endpoint, while further studies are needed to confirm generalizability.

## Conflicts of interest

None disclosed.
